# Endoscopic ultrasonography-guided B3 branch drainage/anastomosis as hepaticoduodenostomy in atrophic left hepatic lobe case

**DOI:** 10.1055/a-2767-1716

**Published:** 2026-01-28

**Authors:** Kohei Kurihara, Hiroyuki Isayama, Ayane Matsuzaki, Ryunosuke Hakuta, Naminatsu Takahara, Yukiko Ito, Hideo Yoshida

**Affiliations:** 1 26307 Department of Gastroenterology, Japanese Red Cross Medical Center, Tokyo, Japan; 2 Department of Gastroenterology, Graduate School of Medicine, The University of Tokyo, Tokyo, Japan; 3 Department of Gastroenterology, Graduate School of Medicine, Juntendo University, Tokyo, Japan; 4 26683 Division of Gastroenterology, Department of Medicine, Faculty of Medicine, Chulalongkorn University, Bangkok, Thailand; 5 13131 Department of Gastroenterology, Tokyo Womenʼs Medical University, Tokyo, Japan

Endoscopic ultrasonography-guided hepaticogastrostomy (EUS-HGS) is widely performed as a salvage procedure when endoscopic retrograde cholangiopancreatography (ERCP) is difficult or has failed 1 2 . EUS-HGS is the most common type of EUS-guided biliary drainage or anastomosis (EUS-BD/A) for the left intrahepatic bile duct (IHBD), which is punctured from the stomach 3 . In contrast, EUS-guided hepaticoduodenostomy (EUS-HDS) is usually performed by approaching the right IHBD from the duodenum 4 . However, we report a case of EUS-HDS approaching the left IHBD in a patient with an atrophic left hepatic lobe.

**Fig. 1:**
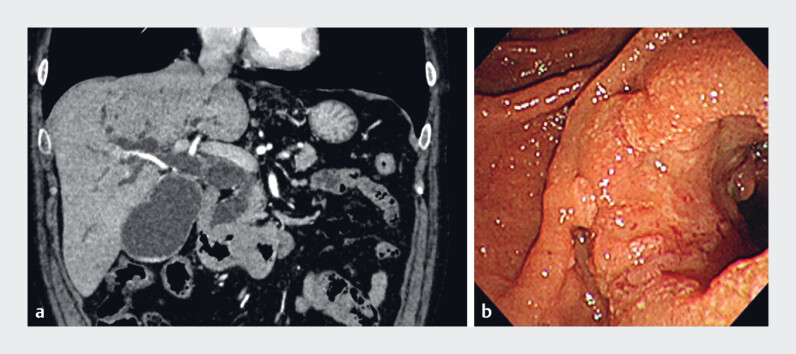
**a** Computed tomography showing wall thickening of the duodenum and dilation of the common bile duct and intrahepatic bile ducts. **b** An endoscopic image showing circumferential wall thickening of the descending duodenum. The tumor involves major papilla.

**Fig. 2:**
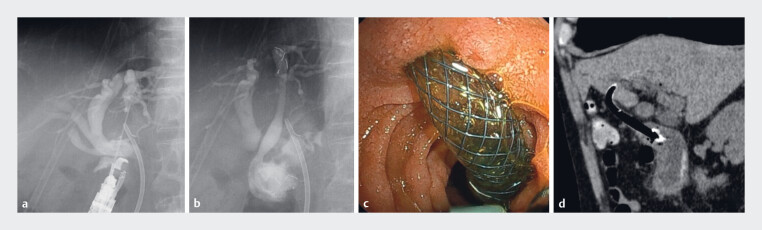
**a** A fluoroscopic image showing puncture and opacification of the B3 using a 19-gauge needle through the echoendoscope. **b** A partially covered self‐expandable metallic stent (SEMS) with an antimigration system was deployed between the B3 and the duodenum. **c** An endoscopic view showing the SEMS in the duodenum with an adequate intraduodenal portion. **d** A sagittal computed tomography image confirming the appropriate positioning of the metallic stent without bile leakage.

A 76-year-old man with distal biliary obstruction due to duodenal cancer was hospitalized ( Fig. 1 ). The patient was unfit for surgery because of severe obstructive pulmonary dysfunction, and a 7-Fr straight biliary plastic stent (PS) was placed transpapillary. When the PS became occluded, reapproaching the papilla was unsuccessful because of duodenal obstruction. EUS-HGS was attempted, but the liver could not be visualized from the stomach due to atrophy of the left hepatic lobe. A slightly dilated B3 branch (approximately 2 mm) was visualized from the first portion of the duodenum using a convex-type echoendoscope (EG-740UT, Fujifilm Corp.,Tokyo, Japan) 5 , and puncture was performed with a 19-gauge needle (EZ Shot 3 Plus, Olympus, Tokyo, Japan). After successful puncture and cholangiogram, a 0.025-inch guidewire (VisiGlide2, Olympus, Tokyo, Japan) was inserted. The tract was dilated using a drill (Tornus ES; Asahi Intec, Aichi, Japan) and a 4-mm balloon dilator (REN; Kaneka, Osaka, Japan). Finally, a partially covered self-expandable metallic stent with an antimigration system (8 mm×12 cm Spring Stopper; Taewoong Medical, Seoul, Korea) was deployed ( Fig. 2 ; Video 1 ). Post‐procedural computed tomography confirmed appropriate stent placement and no adverse events were observed.

Endoscopic ultrasound-guided drainage/anastomosis of the B3 as hepaticoduodenostomy.Video 1

In standard practice, EUS-HDS targets the right IHBD from the first portion of the duodenum. However, in cases with an atrophic left hepatic lobe, EUS-HDS targeting the left IHBD may also represent a feasible option for EUS-BD/A.

Endoscopy_UCTN_Code_TTT_1AS_2AD
